# Factors related to the dangerousness of psychiatric inpatients

**DOI:** 10.1192/j.eurpsy.2021.1017

**Published:** 2021-08-13

**Authors:** M. Kacem, S. Khouadja, S. Brahim, A. Chaouch, L. Zarrouk

**Affiliations:** 1 Department Of Psychiatry, University hospital of mahdia, chebba, Tunisia; 2 Psychiatry, University Hospital Of Mahdia, Mahdia, Tunisia; 3 Department Of Psychiatry, University Hospital of Mahdia, chebba, Tunisia; 4 Department Of Psychiatry, University Hospital Of Mahdia, Tunisia., Psychiatry, Mahdia, Tunisia

**Keywords:** dangerousness, factors, psychiatric, inpatients

## Abstract

**Introduction:**

Mental illness may explain some acting outs, but it does not necessarily lead to a dangerous attitude.

**Objectives:**

Describe the socio-demographic, clinical and therapeutic characteristics of patients considered dangerous and to identify the determinants of psychiatric dangerousness.

**Methods:**

We carried out a descriptive and analytical cross-sectional study during six months including patients hospitalized in the psychiatric department at the University Hospital of Mahdia. The data was collected using a 47-item pre-established questionnaire. The assessment of general psychopathology was carried out using the Brief Psychiatric Rating Scale (BPRS) and that of dangerousness using the Historical Clinical Risk-20 scale (HCR-20).

**Results:**

We have collected 143 patients. The average age was 35 years. The majority of patients were single (70.6%). More than half of the population had addictive behaviors (60.1%). Personal psychiatric and criminal histories were present in 81.1% and 11.9% of cases respectively. More than three-quarters of patients (81.8%) were hospitalized without their consent. Hetero-aggressiveness was the main reason for hospitalization (67.8%). The diagnosis was schizophrenia and bipolar disorder type 2 in 21% of cases for each. The evaluation of psychiatric dangerousness by the HCR-20 scale revealed a mean score of 20.6 with an HCR-20 > 20 in 58.7% of cases indicating a high risk of violence. Factors contributing to violent or criminal behavior in psychiatric inpatients were marital status, presence of personal psychiatric history, presence of criminal history and hospitalization modalities.

**Conclusions:**

The results of our study were generally consistent with the data in the literature.

